# “It’s All We Got Left”. Why Poor Smokers are Less Sensitive to Cigarette Price Increases

**DOI:** 10.3390/ijerph6020608

**Published:** 2009-02-10

**Authors:** Patrick Peretti-Watel, Jean Constance

**Affiliations:** 1 INSERM, U912 (SE4S), IPC, 232 Bd Sainte-Marguerite, 13273 Marseille Cedex 09, France; E-Mail: jean.constance@free.fr(J. C.); 2 Université Aix Marseille, IRD, UMR-S912, 58 Bd Charles Livon, 13284 Marseille Cedex 07, France; 3 ORS PACA, Observatoire Régional de la Santé Provence Alpes Côte d’Azur, 23 rue Stanislas Torrents, 13006 Marseille, France

**Keywords:** Cigarette smoking, poverty, smoking motives

## Abstract

In France, between 2000 and 2008, concurrently to the increase in cigarette price, we observed an increasing social differentiation of cigarette smoking: smoking prevalence decreased among executive managers and professional occupations, it remained stable among manual workers, and it increased among the unemployed. Poor smokers were heavier smokers, they were more frequently tobacco-dependent, and they were more prone to smoke automatically or to reduce “negative feelings”. In-depth interviews provided a more comprehensive insight into poor smokers’ motivations: they were aware of their addiction, but they also talked about the pleasure they get from smoking, and they highlighted the essential needs satisfied by smoking: stress relief, cheap leisure, compensation for loneliness, break-up or redundancy… Acknowledging the functional aspects of smoking experienced by poor smokers helps to understand why increasing the cigarette price is unlikely to deter many poor smokers from smoking.

## 1. Introduction

According to the World Health Organization (WHO), cigarette smoking is the second major cause of death in the world, with about five million deaths each year [1]. Many developed countries have joined the “global war” on smoking launched by the WHO, with extensive tobacco control policies including high cigarette taxes, smoking bans in enclosed public places, restrictions on advertising and selling, as well as public education campaigns and health warnings on cigarette packs. In France, cigarette smoking causes every year about 60,000 premature deaths [2], and the social cost of smoking has been estimated at 1.1% of the Gross Domestic Product [3]. Tobacco control has become a priority for the French government, who strengthened its anti-tobacco policy in the early 2000s. The spearhead of this policy was a sharp increase in cigarette taxes. Indeed, between 2000 and 2008, the French government raised cigarette prices by 66% (from 3.20 € to 5.30 € per pack), and a further increase is planned in 2009. As a result, between 2000 and 2005 the smoking prevalence among the French population declined slightly, from 33% to 30% [4].

Concurrently with the strengthening of tobacco control policies, previous studies conducted in several developed countries concluded that cigarette smoking was increasingly correlated to socioeconomic disadvantage. In the UK, Marsh and McKay found that since the 1970s the smoking prevalence halved among professional and managerial categories, but fell only by one third among unskilled manual workers [5], and another longitudinal study concluded that the social differentiation of smoking increases across the life-course [6]. In other European countries, the smoking prevalence is higher among the least qualified individuals [7], and this relationship has amplified between 1985 and 2000 (especially in Scandinavia, Germany, Italy, Spain) [8,9]. Regarding the USA, Franks *et al.* [10] found that increased real cigarette-pack price over time was associated with a marked decline in smoking among higher-income people, but not among lower-income people. Other studies found that living under the poverty threshold is correlated with smoking, even after adjusting for the effects of several confounders (depression, other substance abuse) [11,12]. Moreover, in both the UK and the USA, poor smokers are claimed to be “poor quitters”: the most deprived smokers attempt to quit as frequently as others, but succeed a lot less frequently [6,13]. These converging results suggest that anti-tobacco policies, after having gained ground in all social environments, are now up against a “hard core” of smokers, less sensitive to price increases, and concentrated in the most underprivileged social strata [10,14]. This increasing association of enduring smoking with markers of social disadvantage has raised concern among public health experts [15].

The aims of the present article were two-fold. Firstly, we studied the social differentiation of cigarette smoking in France between 2000 and 2008, using quantitative data extracted from several national surveys. We hypothesised that during the 2000s the decrease in smoking prevalence was less pronounced among low socioeconomic status populations. Secondly, in order to better understand why poor smokers are less sensitive to price increases, we investigated their smoking habits and motives using both quantitative and qualitative data. Quantitative data extracted from a national representative telephone survey conducted in 2008 were used to compare poor smokers and other smokers’ motives, and in-depth interviews carried out with poor smokers were used to specifically study their smoking motives.

## 2. Material & Methods

### 2.1. Analysis of the Social Differentiation of Smoking, 2000–2008

In order to investigate the social differentiation of cigarette smoking in France, we first used data abstracted from several telephone surveys conducted by the National Institute for Prevention and Health Education (INPES) between 2000 and 2008. These surveys have been carried out with a very similar methodology, among representative samples of French aged 18–75 in 2000 (sample size N=13,685), 2003 (N=3,085), 2005 (N=30,513), 2006 (N=3,206), 2007 (N=6,007) and 2008 (N=2,000) [16]. For each survey, a weighting procedure was computed to improve the sampling: distributions in each sample were adjusted to distributions in the French population for age, gender, occupation, geographic area and size of town (thanks to data available from the 1999 and 2005 French Census).

We used these data to compare the trends in smoking prevalence for three contrasted occupational categories: executive managers and professional occupations, manual workers, and unemployed people. We computed standard z-tests to compare the proportions of smokers across time (for a given occupational category) and across occupational status (for a given year). The trends in smoking prevalence were also compared with the evolution of the standard price of cigarette packs.

### 2.2. Quantitative Analysis of Smoking Motives: Poor Smokers versus Other Smokers

We focused on the last telephone surveyed conducted by the INPES in 2008 among a random sample of 2,000 respondents aged 18–75 because this survey, contrary to previous ones, included questions dealing with smoking motives and smokers’ reactions to the cigarette price increase. Within each selected household, the ‘next birthday’ method was used to choose which member of the household should be asked to participate to the survey (i.e. the investigator asked which person had his birthday closest in the future). If this person was absent or could not respond at once, the investigator proposed a later appointment. In case of refusal, he/she was not replaced by another member of his/her household. The response rate reached 71%. Among the 2,000 respondents, 621 were smokers (for further methodological details on INPES telephone surveys, see [17]).

Respondents were asked about the current financial situation of their household with the following response items: “wealthy”, “satisfying”, “it’s a bit on the short side”, “It’s hard to make both ends meet’, “we had to get into debt”. We considered that those smokers who answered “It’s hard to make both ends meet” or “we had to get into debt” were poor smokers, while other items corresponded to “other smokers”. In order to check the consistency of this categorisation, we first detailed the corresponding sociodemographic profiles: household income level, respondent s educational level and occupational status, being a single parent, as well as gender and age. Then we compared poor smokers and other smokers’ according to their smoking habits (daily consumption of cigarettes, tobacco dependency with the short Fagerström test [18]), their reactions to the cigarette price increase (smoking cheaper or hand-rolled cigarettes, reducing one s cigarette consumption), and their smoking motives (adapted from [19]: the original scale – a 23-item questionnaire – was too long for our study): automatic smoking, aid to socialisation, enjoyment, stress relief, improvement in concentration, to take one’s mind off cares and worries, weight control (respondents were asked to rate each motive on a scale of 1 to 10). A smoker was considered strongly dependent if she/he smoked more than 20 cigarettes per day, or if she/he reported smoking her/his first cigarette within 30 minutes after waking up. Comparisons between poor smokers and other smokers were performed with Pearson s χ^2^ (for categorical variables) and Student’s T (for numerical variables) tests.

### 2.3. Qualitative Analysis of Poor Smokers’ Motives

To define poverty, we endorsed the sociological perspective initially developed by Simmel [20]: the poor person is the individual who receives assistance because of this lack of means. Such perspective was theoretically relevant, but it also provided an easy way to recruit respondents. Indeed, we contacted smokers through social workers who deliver assistance to the poor in two cities of South-Eastern France. In-depth semi-structured interviews were conducted by J.C. in either social service delivery settings or participants’ homes at their convenience.

A brief interview topic guide allowed conversation and meaning to emerge through an interplay between researcher and participant. Interviews lasted between 40 and 90 minutes. Interviews were tape-recorded with informed consent, then transcribed *verbatim* by J.C., with any personal identifying information removed to preserve anonymity and confidentiality (first names have been changed in the quotations *infra*). We endorsed an inductive approach based on grounded theory [21,22]. Data were analyzed concurrently with data collection: the themes emerging from the first five interviews helped to refine the topic guide used for the next five interviews, and so on. J.C. and P.P.W. coded the transcripts independently, then met to discuss their codes and to agree on any differences in language for the codes. Finally, we undertook a second round of coding to condense our set of initial codes into more abstract, second-line codes.

We considered that the sample was saturated after 31 interviews (so data collection ceased), as the last interviews no longer elicited themes not already raised by previous participants. The 31 participants were 13 women and 18 men, seven aged 30 or less, 12 aged 30 to 50, 12 aged 51 to 60. All participants reported financial and/or housing problems, and 25 were currently unemployed. We referred to the analytic methods of grounded theory for guidance concerning the process of undertaking systematic data coding [21,22]. A range of themes emerged from the analysis, but we concentrated below on smoking motives.

## 3. Results

### 3.1. The Social Differentiation of Smoking in France, 2000–2008

**Figure 1.**
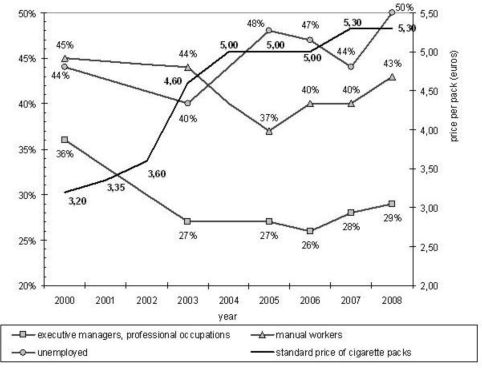
Trends in smoking prevalence for executive managers and professional occupations, manual workers and the unemployed, 2000–2008, France.

Figure 1 displays the trends in smoking prevalence among executive managers and professional occupations, manual workers and the unemployed, as well as the evolution of the standard price of cigarette packs, between 2000 and 2008.

Among the executive managers and the professional occupations, the smoking prevalence declined significantly between 2000 and 2003 (from 36% to 27%, *p*<0.001), concurrently to a sharp increase in cigarette price (from 3.20 € to 4.60 € per pack). Then it remained stable from 2003 to 2008, with no statistically significant variation. Overall, the smoking prevalence decreased by 19% ([36%–29%]/36%=19%) within this occupational category. Among manual workers, there was also a significant decrease in smoking prevalence, but it occurred later, between 2003 and 2005, from 44% to 37% (*p*<0.001). After this decrease, between 2005 and 2008, as the cigarette price only increased slightly, the smoking prevalence among manual workers came back to its initial level (from 37% in 2005 to 43% in 2008, *p*<0.001). Finally, smoking prevalence amongst unemployed people showed a somewhat erratic pattern over time, but it was higher in 2008 (50%) than in 2000 (44%) (*p*<0.001). Moreover, when compared to manual workers and unemployed people, executive managers and professional occupations were significantly less likely to smoke during the whole period, with a widening gap (36% *versus* 44% and 45% in 2000, 29% *versus* 43% and 50% in 2008). Finally, the smoking prevalence was very similar for manual workers and the unemployed in 2000, and it was higher for the unemployed in 2008 (but the difference was not statistically significant, due to the small number of manual workers and unemployed people in the 2008 sample, respectively n=299 and n=98).

This increasing social differentiation of smoking could be partly due to shifts in the socio-demographic composition of occupational categories. Nevertheless, between 2000 and 2008 the mean age did not change within the unemployed group (36 years-old) and among manual workers (38 years-old), and varied only slightly for executive managers and professional occupations (from 41 to 42 years-old). Regarding gender, 82% of manual workers were men in both 2000 and 2008, and this proportion only decreased slightly (from 65% to 62%) among executive managers and professional occupations. Finally, the proportion of men increased among unemployed respondents (41% in 2000, 45% in 2005, 48% in 2008). This shift may contribute to explain some of the rise in smoking prevalence among the unemployed, but it certainly does not explain the whole picture.

### 3.2. Comparing Poor Smokers and Other Smokers’ Habits and Motives

Table I compares the socio-demographic background of “Poor smokers” and “Other smokers” categories, in order to check that the “Poor smokers” category was positively correlated to some indicators of socioeconomic deprivation. Respondents gathered in the “Poor smokers” category were more frequently women than other smokers, with a higher proportion of people aged 35–49 years-old. These poor smokers were also more frequently manual workers or clerks (66% *versus* 47%, *p*<0.001), they were more likely to report that they have not completed high-school (25% *versus* 48%, *p*<0.001), and they were also more likely to report a monthly household income lower than 1,500 € (51% *versus* 86%, *p*<0.001). Finally, 16% of poor smokers were single parents (*versus* 6% among other smokers, *p*<0.01).

Table 2 compares poor smokers and other smokers’ smoking habits, their reactions to the cigarette price increase, as well as their smoking motivations. On average, poor smokers were heavier smokers: 21% smoked more than 20 cigarettes per day (*versus* 12% among other smokers), and 46% were strongly dependent to nicotine (*versus* 28%). Concerning reactions to the cigarette price increase, about one third of poor smokers and other smokers reduced their cigarette consumption, but poor smokers were more likely to turn to cheaper or hand-rolled cigarettes (50% did so, *versus* 33% for other smokers).

Finally, regarding smoking motives, poor smokers were more prone to consider smoking as an automatic activity, or as a means for reducing negative feelings (stress relief, taking one’s mind off cares and worries). Conversely, other smokers were more prone to smoke in order to socialise more easily with others, or to improve their concentration. Moreover, three motives obtained an average score higher then 6 amongst poor smokers (automatic smoking, enjoyment, stress relief), *versus* two motives amongst other smokers (aid to socialisation and enjoyment).

### 3.3. Smoking Motives amongst the Poor: a Qualitative Exploration

During in-depth interviews, several participants stated that tobacco was a “hard drug”, some labelled themselves “addicts”, and most of them acknowledged being tobacco-dependent. Their perception of their own dependence was based on what they could do for continuing smoking despite the cigarette price increase: to deprive themselves of other goods, to beg for cigarettes, or even to pick up butts:

*I need a cigarette every five minutes or I lose my temper.* (…) *I would rather give up coffee, drinking and so on, but no, not smoking. It’s my drug. That’s all.* (…) *I feel it’s the only thing that keeps me going, the only thing. It’s the only company I have.* (...) *Even if the pack costs 50 €, I will buy it. But I don’t have so much money. I will never, never quit.* (Philippe, man aged 50, homeless and unemployed)

*I am unbearable when I feel the urge for a cigarette.* (…) *I feel as if I’m going round in circles. I yell at everyone, my daughter doesn’t even talk to me because she knows I will send her packing. I’m just insufferable. Then I go downstairs in the street to find a cigarette. I ask people.* (…) *That’s the only thing I can beg for. It would never occur to me to beg for money. But a cigarette, sure, no problem*. (Melina, woman aged 54, unemployed)

*When I feel the urge for a cigarette I could smoke butts picked up in the street. I could live without everything but the cigarettes*. (…) *I have been smoking for a long, long time. I’m so hooked to cigarettes. But I would like to quit right now. If only I could quit I would shout it out loud, yeah I would sing it “I quitted! I quitted”* (…) *When I need a fag I get extremely nervous, I search everywhere because sometimes I stash some cigarettes in the kitchen or in the living room, just in case.* (…) *When I inhale smoke it’s a little moment of happiness, something I steal just for me*. (Manon, woman aged 56, clerk)

In the last quotation *supra*, Manon said she is hooked on smoking, she would like to quit, but nevertheless she claimed that every cigarette provided her with “a little moment of happiness”. Several other participants talked about the pleasure and happiness they get from smoking. More generally, these smokers stressed the essential needs satisfied by smoking. First, all participants claimed that smoking relieves their stress. For example, Didier considered that his wife and daughter smoke because of their stressful life, and he felt guilty for not being able to earn enough money to offer them a better life:

*What worries me too is not being able to afford to give my wife and daughter a better life, a different life where they wouldn’t need to smoke like I do. I feel guilty about that. I would like to give them a life with less stress and worry, because – well, I get the minimum wage and we can’t make ends meet. I don’t earn much and we have to struggle.* (…) *My wife can’t give up smoking because of the stress. Poverty and living from day to day gives you nervous tension all the time, which creates a need. Cigarettes meet that need, although they are rather a poor substitute. They give one a little enjoyment. Since people can’t afford anything else, they rely on things like smoking.* (Didier, man aged 53, manual worker)

But a majority of participants (of both genders, and especially the older ones, the unemployed and those living alone) also claimed that cigarette smoking fills a void in their everyday life: they smoke because they have nothing else to do, because it is the only leisure activity they can afford (paradoxically, some smokers believe they save money by smoking), conversely they remembered that they used to smoke less when they had a job; they also smoke because they feel lonely, in order to compensate for an emotional break-up or for redundancy, or to relief withdrawal symptoms after having stopped ‘hard drug’ use. In other words, many interviewed smokers considered that smoking is ‘all they got left’.

*I’m so intoxicated.* (…) *Okay, smoking gives cancer, but it also gives happiness, not a great happiness, but a little bit of it* (…) *I need my cigarettes because I don’t have spare-time activities, I’m broke, I don’t get out, I don’t go to the movies* (…) *There’s nothing here, we are miles from everywhere and there’s nothing to do. This place is dead and so are we. We should use our energy on other things than smoking and all that shit.* (...) *What do you think there is to do here? Going down to the pub? Meeting the same people, repeating the same bullshit, smoking cigarette after cigarette?* (…) *We’re like zombies here because we’re miles from everywhere. Look, a swimming pool, it would be great to have a pool nearby... If there was a pool, I would go swimming every day. Instead of smoking, I would go swimming, for example. It may sound stupid, but it’s not that stupid, actually* (...) *I haven’t got a car, and I couldn’t afford the petrol anyway* (...) *I’m not going to hitch a lift to the swimming pool, am I?* (Camille, woman aged 60, retired)

*Smoking, yes it costs me more than 100 € a month... That’s a lot. It makes the budget rather tight, but thanks to my partner’s job, we practically manage to get to the end of the month – some months a least. Anyhow, cigarettes are a definite item, there’s no arguing about that.* (…) *I would like to stop smoking – um, because of the dough – to do other things with all the dough we spend on cigarettes. But it’s not easy. It’s all we have left, since we’ve stopped going out and we’ve given up booze. It’s our only pleasure these days.* (Melina, woman aged 54, unemployed) *If you’ve got no money, you just stay at home and don’t budge. Smoking is my only relaxation. Going places, eating out, having fun is too expensive. Look at the people who go to football and rugby matches. That costs as much as a pack of cigarettes, if not two or three times more.* (…) *Plus the cost of getting there. Smoking is all we have! It’s our only way of relaxing.* (Joseph, man age 50, unemployed)

*When I used to work for a building firm, I smoked less. But now it’s hard to find a job.* (…) *Smoking helps me. When I wake up in the night I can’t sleep anymore. So I smoke. What else to do? Nothing. I smoke. And if I ain’t got cigarettes, then… That’s bad!* (…) *There is also loneliness. Loneliness makes you smoke.* (Clement, man aged 51, unemployed)

*Smoking. Sometimes I tell myself it’s all I have. Why? Because there was a big upheaval in my life. My smoking increased because of that upheaval* (...) *I was smoking already, but the upheaval made it worse: suddenly being myself on my own. Cigarettes keep me company. If I haven’t got any, I feel a craving, and they do actually keep me company. They are there, they are there. That’s it. When I smoke I’m less alone. Well I mean... it’s hard to admit, but I feel less lonely.* (Solange, woman aged 49, unemployed)

*I started smoking when I stopped cocaine.* (…) *I restarted smoking when I lost my job and found myself out on the street.* (…) *I had no income so I cadged off people. Smoking calmed me down.* (Karine, woman aged 28, unemployed)

## 4. Discussion

In France, among executive managers and the professional occupations, the prevalence of smoking declined significantly between 2000 and 2003, then it remained stable till 2008. Among manual workers, the decrease occurred later, was less pronounced, and after 2005 the smoking prevalence came back to its initial level. Among unemployed people, the smoking prevalence exhibited a more erratic pattern over time, and it was higher in 2008 than in 2000. These trends clearly demonstrate that since 2000 there is an increasing social differentiation of cigarette smoking in France. Regarding smoking habits, poor smokers were heavier smokers, they were more frequently tobacco-dependent, and they more frequently react to the cigarette price increase by turning to cheaper or hand-rolled cigarettes. Regarding smoking motives, poor smokers were more prone to smoke automatically, or for reducing negative feelings, and less prone to smoke to socialise or improve concentration. Finally, in-depth interview showed that poor smokers were aware of their addiction to nicotine. But they also talked about the pleasure and happiness they get from smoking, and they highlighted the essential needs satisfied by smoking: stress relief, cheap leisure, a compensation for loneliness, break-up or redundancy… They also frequently claimed that smoking is “all they got left”.

Several limitations of the present study must be acknowledged before discussing our results. Firstly, repeated cross-sectional data provide smoking rates to compare one with another, but each rate mixes lifetime consumption paths observed at various stages for successive birth cohorts. A life-course perspective could have better captured the dynamics of smoking [23,24]. Secondly, changes in smoking prevalence result from changes in both smoking initiation and cessation. Nevertheless, previous analyses based on the same data showed that the social differentiation of smoking is mostly due to contrasted quitting rates [4]. Concerning poor smokers and other smokers’ habits and motives, data were collected with a closed-ended questionnaire that prevents respondents from qualifying or justifying their responses, and we lack detailed information about non-respondents. Conversely, qualitative data provided a more comprehensive insight into poor smokers’ perspective, but corresponding results cannot be generalised. Finally, the qualitative and the quantitative surveys did not use the same definition of poverty. In our quantitative study we opted for a subjective measure of poverty, because an objective one would have been to simplistic, unless we had much more information on respondents’ living conditions. Nevertheless, our self-report measure was consistent with some objective indicators (occupation and monthly household income, see Table 1).

We consider that it is very important to understand why people, and especially poor people, keep on smoking cigarettes despite extensive anti-tobacco policies, including cigarette price increases. Indeed, the scientific understanding of smoking behaviours is currently dominated by a biomedical model that tends to overlook smokers’ motives, because within this model people simply smoke to reverse the symptoms of nicotine withdrawal [15,25]. Our quantitative results suggest that poor smokers are less likely to quit than wealthier smokers because they are more frequently tobacco-dependent, and because they satisfy more essential needs with cigarette smoking (the reduction of negative feelings, instead of facilitating socialisation or improving concentration). This interpretation is partially confirmed by our qualitative data, as most participants reported smoking to relief stress or to compensate for boredom or loneliness. Nevertheless, in-depth interviews with poor smokers also suggest to complete and to qualify this interpretation.

Firstly, in the quantitative analysis the “enjoyment” motive reached similar levels among poor and other smokers. But in-depth interviews reveal that the pleasure poor smokers derived from smoking is quite specific: they frequently considered cigarette smoking as the only pleasure they can afford, as the one thing that offers them “a little moment of happiness”. This is probably not the same pleasure among wealthier smokers. Moreover, according to the common understanding of addiction, dependent people just take their drug because they need it to relief withdrawal symptoms. In other words, they need to smoke because they are dependent. But that is not what interviewed smokers said. On the contrary, from their point of view, they are rather tobacco-dependent because they need cigarettes to satisfy essential needs that cannot be met otherwise, because of their deprived condition. This remark echoes a previous qualitative study conducted by Jason Hughes in the United Kingdom [26]. According to Hughes, some smokers endorse a dual conception of dependence. On the one hand, they admit to be enslaved by cigarettes. On the other hand, the meaning of dependence seems to involve the idea that tobacco is something that smokers can depend upon, a reassuring emotional resource that will always be there for them.

Finally, our findings suggest that harsh living conditions generate socioeconomic stress that drives poor smokers to keep on smoking tobacco. Previous qualitative studies support this interpretation [27,28]. Conversely, other studies suggest that smoking relapse frequently occurs after divorce or redundancy, while successful cessation is associated with positive life changes [5,29]. Thus poor smokers may be less successful in quitting smoking because they lack such positive changes in their existence. Nevertheless, one could assume that using tobacco to manage stress is a culturally shaped behaviour. In other words, poor smokers may be more prone to report such use not only because they endure more stress than wealthier smokers, but also because they have learned to do so during their socialisation. Indeed, smoking used to be a deep-rooted and highly valued habit within the working-class culture [30]. Besides, during the 1970s, French trade unions fiercely opposed the increase in cigarette prices, claiming that cigarettes were a legitimate antidote to the strains endured by workers. This assumption is also supported by the fact that some interviewed smokers explicitly linked their smoking habit to their harsh living conditions (see Didier s quotation *supra*). We believe this topic deserves further investigation. More generally, further research is needed to better understand the cultural shaping of smoking motives.

## 5. Conclusions

In France as in many other developed countries, there is an increasing social differentiation of cigarette smoking. Despite a sharp increase in cigarette price during the early 2000s, the smoking prevalence barely decreased among manual workers and even increased among unemployed people. Acknowledging the attractive and pleasurable aspects of smoking experienced by poor smokers helps to understand why anti-tobacco policies in general, and the increase in cigarette price in particular, are unlikely to deter many poor smokers from smoking. Moreover, raising cigarette prices is likely to impose a disproportionate burden on those poor smokers who do not quit. Non-smoking specific interventions that would contribute to improve poor smokers’ living conditions (for example, facilitating their access to leisure activities and re-weaving the social fabric in their neighborhood, to reduce their feelings of idleness and loneliness), as well as the conception and promotion of alternate strategies for coping with “socioeconomic stress”, might be more effective to promote cessation.

## Figures and Tables

**Table 1.** Socio-demographic profile of poor smokers and other smokers, France, 2008.

	Poor smokers (N=115)	Other smokers (N=506)
	*row percentages*	
Gender		
-women	56%	44%
-men	44%	56% *
Age (in years)		
-18–24	10%	20%
-25–34	29%	27%
35–49	40%	32%
50–64	20%	17%
65–75	1%	4% #
Occupation:		
-manual worker / clerk	66%	47%
-other	34%	53% ***
Educational level:		
-< high-school	75%	52%
-≥ high-school	25%	48% ***
Monthly household income: †		
-< 1,500 €	49%	14%
- ≥ 1,500 €	51%	86% ***
Single parent:		
-no	84%	94%
-yes	16%	6% **

***,**,*,#, ns: respectively significant at p<0.001, 0.01, 0.05, 0.1, non significant (Pearson’s χ^2^).

† 41 smokers did not reported their income level and where excluded from the bivariate analysis.

**Table 2.** Smoking behaviours and motives of poor smokers and other smokers, France, 2008.

	Poor smokers (N=115)	Other smokers (N=506)
	*row percentages and means*	
Daily cigarette consumption		
- 1 to 5 cigarettes	12%	20%
- 6 to 10 cigarettes	35%	33%
- 11 to 20 cigarettes	32%	35%
- > 20 cigarettes	21%	12% *
Tobacco dependency (Fagerström):		
- none / mild / moderate	54%	72%
- strong	46%	28% ***
Smoking less cigarettes since the cigarette price increase		
-no	63%	66%
-yes	37%	34% ns
Smoking cheaper or hand-rolled cigarettes since the cigarette price increase		
-no	50%	67%
-yes	50%	33% ***
Smoking motives (scale of 1 to 10):		
-automatic smoking	6.7	5.2 ***
-aid to socialisation	4.4	6.9 ***
-enjoyment	6.7	6.4 ns
-stress relief	6.4	5.8 #
-improvement in concentration	1.9	2.6 *
-to take one’s mind off cares and worries	4.4	3.7 #
-weight control	2.1	2.2 ns

***,**,*,#, ns: respectively significant at p<0.001, 0.01, 0.05, 0.1, non significant (Pearson s χ^2^ and Student s T).
